# Individual differences could explain the failure in transitive inference formation in pigeons using probabilistic reinforcement

**DOI:** 10.3389/fpsyg.2022.1033583

**Published:** 2023-01-17

**Authors:** Héctor Octavio Camarena, Oscar García-Leal, Zayra Saldaña-Hernández, Erick Barrón

**Affiliations:** ^1^Department of Environmental Sciences, University of Guadalajara, Guadalajara, Mexico; ^2^Basic Psychology Department, University of Guadalajara, Guadalajara, Mexico

**Keywords:** Pavlovian mechanisms, reasoning process, probabilistic reinforcement, transitive inference, task complexity

## Abstract

In propositional logic, it is stated that “for if A is predicated for every B, and B for every C, A must necessarily be predicated of every C”. Following a similar logical process, it can be said that If A > B and B > C, then A > C, this is called transitive inference (TI). Piaget developed a verbal task to evaluate TI in children. Subsequent studies adapted this task for animals using a conditioned discrimination between five-terms sequence of stimuli A + B-, B + C-, C + D-, and D + E-. If subjects prefer B over D during test, it is assumed that TI has occurred. In this experiment, we analyzed the effects of task complexity on TI by using a five-terms sequence of stimuli associated with probabilistic outcomes during training, in pigeons. Thus, both stimuli are reinforced in each pair but with different probability, 0.8 for + stimulus and 0.2 for the—stimulus. We found that performance during C + D- pair is impaired and preference in the test pair BD is affected. However, this impairment is dependent on individual differences in performance in C + D- pair. We compare our findings with previous research and conclude that Pavlovian mechanisms, as well as ordering of stimuli, can account for our findings.

## Introduction

In propositional logic, it is stated that “for if A is predicated for every B, and B for every C, A must necessarily be predicated of every C” (Aristotle, 1889 trans). Following a similar logical process, it can be said that If A > B and B > C, then A > C, this is called transitive inference (TI).

Transitive inference (TI) is usually defined as the ability to “deduce” an untrained relationship between stimuli that are previously trained. This relation follows a hierarchy with the form: if A > B > C > D > E, then B > D. This ability was first experimentally studied with verbal tasks by Piaget (1921) and found that children below and above 7 years old are incapable of deducing a relation if A > B > C, then A > C, apparently because of a child judgment state called *anesthésie de la compréhension.*^1^ Afterward, Bryant and Trabasso (1971) designed a non-verbal task that showed that with proper training and feedback, children below 7 years are capable to deduce a relation if A > B > C > D > E, then B > D. A few years later, McGonigle and Chalmers (1977) employed an adaptation for monkeys of the procedure Bryant and Trabasso (1971), finding that their subjects were capable of TI even with performance levels similar to humans. Regarding the above findings from humans and animals, different accounts for TI have been proposed (Delius and Siemann, 1998; Vasconcelos, 2008; Gazes and Lazareva, 2021).

Transitive inference usually involves the presentation of simultaneous discrimination between stimuli. So, S + refers to the stimulus always associated with reinforcement delivery, whereas S- is associated with the absence of reinforcement. With this arrangement, five overlapped simultaneous discrimination is trained, where the first stimulus is always reinforced and the last stimulus is never reinforced (A + B-, B + C-, C + D-, D + E-), those pairs are referred to as adjacent pairs. Performance for adjacent pairs usually takes the form of an asymmetric U shape, where extreme pairs are better solved than central pairs, an effect known as the serial position effect (SPE).

After training, the test requires the exposition to the non-adjacent pairs (BD, AC, AD, CE, BE, and AE), where the preference of B over D reaching over chance levels is assumed as proof of TI, whereas the performance of the other non-adjacent pairs is analyzed as a way to evaluate the symbolic distance effect (SDE). SDE implies that non-adjacent stimulus pairs with more intervening elements (e.g., AE) are better solved than those with lesser intervening elements (e.g., BD). Therefore, the performance during the test usually takes the form of an ascending function departing from BD and reaching asymptotic levels at AE.

At least two associative accounts of TI can be addressed. The first one assumes that the direct reinforcement received during training can predict TI during the test. According to this account, during A + B-, fewer errors will be made because both stimuli are neutral (with no previous reinforcement story); however, there will be more errors during B + C- (C responses). This asymmetry in errors would be sufficient to confer more associative strength for B than for C. The same process would produce more errors in C + D- discrimination than in C responses in B + C-. Therefore, the required value ordering for TI can be obtained (Wynne et al., 1992). The second one regards that TI can be predicted from training because of a value transfer mechanism from the positive stimuli to the negative stimuli. Thus, in A + B-, B receives associative strength from an always reinforced stimulus, whereas in D + E-, D does not receive extra associative strength. If B and D are both partially reinforced (A + B- B + C- and C + D-D + E-), the preference of B over D would be a consequence of the differences in value transfer received by each stimulus, since D received less value transfer (von Fersen et al., 1991).

Seeking for the mechanisms involved in TI, several manipulations have demonstrated the conditions that allow, impair or impede TI. For example, Roberts and Phelps (1994) showed that a physical circular arrangement impedes TI in rats, whereas a physically linear arrangement allows TI, supporting the spatial coding hypothesis. Using a different approach von Fersen et al. (1991) demonstrated that a closed series (e.g., X + A-, A + B-, B + C-, C + D, D + E-, F + X-) impeded TI in pigeons. However, their explanation involves the induced changes in value transfer by making X + at the beginning and X- at the end of the series, which would reject the spatial coding hypothesis. More recently, Lazareva et al. (2004) showed that the use of ordered feedback allowed TI in hooded crows, whereas the constant feedback impeded TI. Other studies have focused on deliberately preventing B over D preference by affecting associative strength, for example, Lazareva and Wasserman (2012) employed pigeons as subjects and a bias reversal procedure with massive presentations of D + E-, in order to provoke a higher reinforcement ratio for D, and consequently revert the expected B over D preference. Despite their bias reversal procedure, Lazareva and Wasserman (2012) found a B over D preference with over chance levels. In a more recent experiment, Camarena et al. (2018) found TI in pigeons, despite lower associative strength in B + than in D +, after overtraining in C + D- pair. These findings suggest that associative accounts of TI require further examination.

A recent review of associative models suggests that associative mechanisms might explain the behavior in the TI task, but there are other mechanisms involved (Lazareva et al., 2020; Gazes and Lazareva, 2021). Therefore, the associative account has not been conclusively rejected and relational mechanisms (e.g., the positional ordering of the stimuli) still seem to be involved. In order to further explore the associative account, we employed a new experimental manipulation that increases the complexity of the task but keeps the effects of associative mechanisms. Other studies have employed probabilistic reinforcement in specific premises as a way to evaluate value transfer between stimuli (Zentall and Sherburne, 1994; Weaver et al., 1997). More recent approaches have studied the effect of probabilistic reinforcement on the formation of superstitious learning using TI procedures (Jin et al., 2022). In the present experiment, we manipulated probabilities in all premises, as a way to increase the difficulty of the task and observe its effects on test performance.

This experiment aimed to determine whether the use of probabilistic reinforcement in all premises allows transitive inference in pigeons. The main hypothesis is that the use of probabilistic outcomes should not impair TI, since the general conditions for TI are preserved. However, if relational mechanisms are involved TI would be impaired.

## Materials and methods

### Subjects

The experiment began with eight pigeons, of which six pigeons were experimentally naïve, whereas two had previous experience in a temporal discrimination task. Because of a complete lack of response during auto-shaping one pigeon was excluded [the one with previous experimental experience, resulting in a final sample of seven pigeons (only one of them with previous experimental experience)]. All pigeons were maintained at approximately 70% of their free-feeding body weight. Water was always available throughout the experiment in the individual home cages. All subjects were individually housed (25 cm × 25 cm × 30 cm) and exposed to a 12 h:12 h light/dark cycle, with lights on from 7:00 to 19:00 h.

### Apparatus

Four operant conditioning chambers were used (MED Associates, Inc., Model ENV-018MD). The boxes were 31 cm high, 24 cm long, and 31 cm wide. The front panel was divided into three columns. A 5.5 cm × 6 cm feeder opening, located 3 cm above the grid floor, gave access to the food when the hopper was activated and illuminated by a 2.8 w light. Over the feeder, placed 22 cm above the grid floor, there was a 2.54-cm diameter white cue key. The side columns were equipped with 2.54 cm diameter keys, also placed 22 cm above the grid floor. The three keys were 9 cm apart, center-to-center. The side keys could be illuminated in different colors. The 2.8 w house light was centrally located in the rear panel of the chamber, 27 cm above the grid floor. Each cage was located inside a sound isolated panel (ENV-018V), equipped with a fan that circulated air and masked extraneous noises.

The experimental procedure was approved by the local Ethical Committee of the Center for Studies and Investigations in Behavior, by the University of Guadalajara committee for animal experiments, and met governmental guidelines.

### Procedure

Each session started with the illumination of the house light and the illumination of the central white key. A single peck in the center key turned it off and turned on the two side keys. The color of the side keys depended on the stimuli pair presented, so that A, B, C, D, and E were always associated with red, green, blue, yellow, and cyan cues, respectively, for all the pigeons. The probability of reinforcing each key was eight out of 10 trials for the “ + ” stimulus (*p* = 0.8) and two out of 10 trials for the “-” stimulus (*p* = 0.2). Both probabilities were independent and were controlled by random sampling without replacing. The position of each cue was counterbalanced across trials during the entire experiment. Pecking one of the side keys resulted in turning off both of them, as well as the house light. Pecking the side keys also controlled feeder activation depending on whether the trial was reinforced or not. When the choice was reinforced, the feeder was illuminated, and 4 s of food access were allowed. Immediately, a 10 s inter-trial-interval -ITI- started. When the choice was not reinforced, the side keys and the house light was turned off and a 14 s ITI started. The following trial began with the illumination of the houselight and the illumination (and activation) of the central key as a white cue. All training sessions lasted until 200 trials of training were completed or 1 h had elapsed. Following our previous procedure, we did not use correction trials (Camarena et al., 2018).

Pigeons were trained in four simple overlapped item pairs, A + B-, B + C-, C + D-, and D + E-, based on the same procedure employed in Camarena et al. (2018). So, in each pair, the choice of stimulus + was always followed by the reinforcer, but the choice of stimulus—was always followed by the absence of the reinforcer. Training phases were intended for learning each premise, first individually and then presenting all premises in random order. Test 1 and test 2 were intended for evaluating the effects of overtraining. Since, C + D- is usually the worst solved pair, overtraining was intended for correcting deficits during that pair. The relevant similitudes and differences in results will be mentioned in the discussion.

The training was divided into four different phases. Each phase comprised 200 trials of different types, depending on the phase. In phase 1, only pair A + B- was trained. In phase 2, the pairs A + B- and B + C- were trained, in random order. In phase 3, only the pair C + D- was trained. For these three phases at least an average of 80% of correct responses in two consecutive sessions was required to move from one phase to the next one. In cases where more than one premise was presented (e.g. phase 2 and phase 4), the averaged performance of all premises was regarded in order to compare to the criterion performance value, in the case of phase 2 B + percentage and C + percentage were averaged, whereas in phase 4, percentages from A +, B +, C +, and D + were averaged. Only one exception to the criterion was allowed, if the average correct responses during five consecutive sessions remained below 50%, the next phase was administered regardless of the low performance. This criterion was followed as a way to avoid a positive stimulus being regarded as negative, and vice versa. The exception was employed, since in a previous experiment (unpublished data), we found a drop in performance that could not be corrected, even after several sessions. A similar criterion can be found in the study by Gazes et al. (2014). Training finished after nine more sessions during which pigeons were exposed to all the adjacent pairs: A + B, B + C-, C + D-, and D + E- in random order. These nine sessions were intended to correct any performance deficits from previous phases. Therefore, any subject could reach the criterion in phase 4 by averaging at least 80% of correct responses regarding the obtained percentages of all premises (A + B-, B + C-, C + D-, and D + E-), for two consecutive sessions.

Test 1 comprised 200 trials of non-adjacent pairs BD, AC, AD, CE, BE, and AE, presented in random order and under non-differential reinforcement (*p* = 0.5 for all stimuli). The first test lasted 4 sessions.

Overtraining comprised two more phases. During the first overtraining phase (phase 5), the pair C + D- was once again trained in 400 trials (two sessions). Phase 6 was equal to phase 4, but it lasted only two sessions.

Finally, the second test was administered, it was the same as test 1, but it comprised only a single session (see Table 1).

**TABLE 1:** Phases of the experiment.

	Training				Test 1	Overtraining		Test 2
	Phase 1	Phase 2	Phase 3	Phase 4		Phase 5	Phase 6	
Stimuli	A + B-	A + B- B + C-	C + D-	A + B- B + C- C + D- D + E-	B D A C A D C E B E A E	C + D-	A + B- B + C- C + D- D + E-	B D A C A D C E B E A E
Sessions	80% of correct responses in 2 consecutive sessions or 5 consecutive sessions below 50%			9	4	2	2	1
Trials by Session	200	200	200	200	200	200	200	200

### Data analysis

The average amount of sessions until reaching the criterion was compared between phases. The mean percentage of correct responses for each premise was compared in phases 4, 6, and both tests. For phase 4, the last two sessions were taken, in cases where subjects reached the criterion in phase 4, their last two sessions were taken. For test 1, all sessions were regarded as averaging performance for each subject, whereas for test 2, the single session administered was regarded.

Due to the number of subjects, non-parametric statistics were employed. For repeated measures comparisons, the Friedman test was employed along with Conover’s *post-hoc* tests. For differences from chance, the Wilcoxon sign rank test was applied. Friedman test was used because it employs ranks, which makes it less sensitive to outliers (Pereira et al., 2015). We regard this feature as suitable for group analyses, particularly when noticeable individual differences are found. For this same reason, the Wilcoxon sign rank test was used. *Conover test* was employed regarding it has similar power as the Friedman test controlling type I error (Al-Subaihi, 2000). This was also suitable for comparing differences from chance since we tried to avoid reporting false positives, particularly for the BD pair.

## Results

**FIGURE 1:**
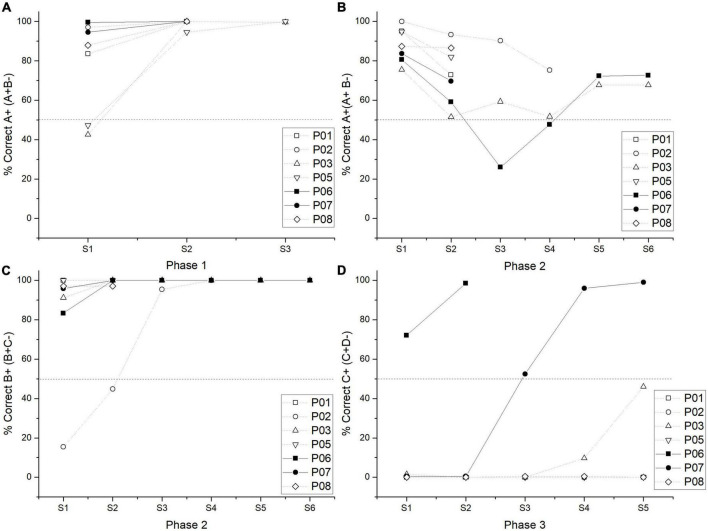
Individual performance in the percentage of correct responses by a session in phase 1 **(A)**, phase 2 for A + stimulus **(B)**, phase 2 for B + **(C)**, and phase 3 **(D)**. Pigeons P06 and P07 were plotted in a straight line since these were the only ones who reached the criterion during phase 3.

The number of sessions to reach the criterion followed an ascending trend from session one to session three (phase 1 *M* = 2.28 *SD* = 0.18; phase 2 *M* = 3.57, *SD* = 0.68; phase 3 *M* = 4, *SD* = 1). However, there were no statically significant differences between phases [χ^2^ (2) = 2, *p* = 0.36]. From phase 1 to phase 2, there was an increase in the variability for individual performance. Consequently, for some subjects, it took two sessions to reach the criterion, whereas, for other subjects, it took six sessions (see Figures 1A–C). The range of sessions for reaching the criterion was three sessions in phase 1, six in phase 2, and five in phase 3.

As expected, phase 3 was the most difficult to solve, so that, group performance averaged 32.81% of correct responses in the last two sessions. Only two subjects reached the criterion, for one of them (subject P06), it took two sessions, and for the other (subject P07), it took five sessions, obtaining an average performance of 98.37% (see Figure 1D). Those who did not reach the criterion averaged only 5.62% of correct responses, regarding their last two sessions. It is worth mentioning that one subject (subject P03) had 46% of correct responses in its last session of phase 3. Therefore, most subjects sustained a floor effect during phase 3 (see Figure 1D).

During phase 4, the low performance in C + D- pair remained so it was the worst solved pair among all subjects. Only two subjects reached the criterion during phase 4 with an average performance of 81.58% (P03 and P07). For one subject (P03), it took three sessions to reach the criterion, for the other (P07), it took six sessions (the one that passed phase 3). For those two subjects, their performance during their last two sessions was taken for statistical analysis.

**FIGURE 2:**
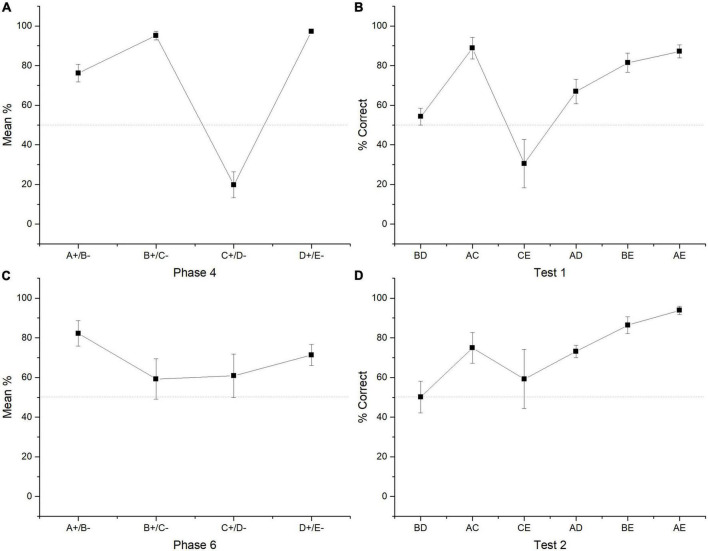
Mean percentage (SEM ±) of correct responses for each premise in the last two sessions of phase 4 **(A)**. Mean percentage (SEM ±) of correct responses regarding all four sessions of test 2 **(B)**. Mean percentage (SEM ±) of correct responses during phase 6 **(C)**. Mean percentage of correct responses during test 2 **(D)**.

Friedman test revealed differences between premises [χ^2^ (3) = 31.791, *p* < 0.001]. *Post hoc* comparisons showed that A + (*M* = 76.23) differed from B + (*M* = 95.16) [*Conover test* (39) = 2.078, *p* = 0.044], from C + (*M* = 19.11) [*Conover test* (39) = 2.820, *p* = 0.008], but did not from D + [*Conover test* (39) = 1.929, *p* = 0.061]. B + differed from C + [*Conover test* (39) = 4.897, *p* < 0.001], but did not differ from D + (*M* = 97.17) [*Conover test* (39) = 0.148, *p* = 0.883]. C + differed from D + [*Conover test* (39) = 4.749, *p* < 0.001]. All correct stimuli reached levels above chance (A +, *p* = 0.001; B +, *p* < 0.001, D +, *p* = 0.001), whereas C + remained below chance (*p* = 0.002) (see Figure 2A).

The four sessions of test 1 revealed that group performance did not differ from chance in the crucial pair BD (*M* = 54.27) (*p* = 0.578) as well as CE (*M* = 30.48, *p* = 0.219). All the remaining non-adjacent pairs differed from chance (AC, *M* = 88.81, *p* = 0.022; AD, *M* = 66.92, *p* ≤ 0.001, BE, M = 81.47, *p* < 0.001; AE, M = 87.17, *p* = 0.016). Friedman test revealed statistically significant differences between premises [χ^2^ (5) = 24.143, *p* < 0.001]. *Post hoc* comparisons showed that BD differed from AC [*Conover test* (30) = 3.157, *p* = 0.004], from BE [*Conover test* (30) = 2.059, *p* = 0.048], and from AE [*Conover test* (30) = 2.882, *p* = 0.007]. AC differed from CE [*Conover test* (30) = 3.569, *p* = 0.001], from AD [*Conover test* (30) = 2.196, *p* = 0.036]. CE differed from BE [*Conover test* (30) = 2.471, *p* = 0.019] and from AE [*Conover test* (30) = 3.294, *p* = 0.003] (see Figure 2B).

During phase 5, subjects marginally improved their performance, so that the average performance reached for C + was 54.96% being not different from chance (*Wilcoxon Z* = 49.500, *p* = 0.875). During phase 6, when subjects were presented with all premises again, the found pattern of phase 4 was distorted. So, C + performance improved but the stimulus pairs where C appears were also affected (e.g., B + C- and D + E-). During phase 6 only stimulus A + (*M* = 82.22) and D + (*M* = 71.35) reached performance levels above chance (A + *Wilcoxon Z* = 95, *p* = 0.008; D + *Wilcoxon Z* = 98, *p* = 0.005), whereas B + (*M* = 59.22) and C + (*M* = 60.82) did not differ from chance (B +, *p* = 0.232; C + *p* = 0.777). Friedman test revealed that there were no statistically significant differences between premises [χ^2^ (3) = 5.743, *p* = 0.125]. However, *post hoc* comparisons showed that only stimulus A + differed marginally from C + [*Conover test* (39) = 2.030, *p* = 0.049] (see Figure 2C).

Performance during test 2 showed that again the critical pair BD (*M* = 50.14) did not differ from the chance (*Wilcoxon Z* = 17, *p* = 0.688), as well as CE (*M* = 59.25, *p* = 0.73) pair (*Wilcoxon Z* = 16.5, *p* = 0.735), whereas all other non-adjacent pairs differed from chance (AC, *M* = 74.91, *p* = 0.034; AD, *M* = 73.15, *p* = 0.016; BE, *M* = 86.35, *p* = 0.016, AE, *M* = 93.82, *p* = 0.022). Friedman test showed differences between premises [χ^2^ (5) = 16.872, *p* < 0.005]. So, BD differed from BE [*Conover test* (30) = 2.619, *p* = 0.014] and AE [*Conover test* (30) = 3.584, *p* = 0.001]. AD differed from AE [*Conover test* (30) = 2.481, *p* = 0.019] and CE differed from AE [*Conover test* (30) = 2.275, *p* = 0.030] (see Figure 2D).

**FIGURE 3:**
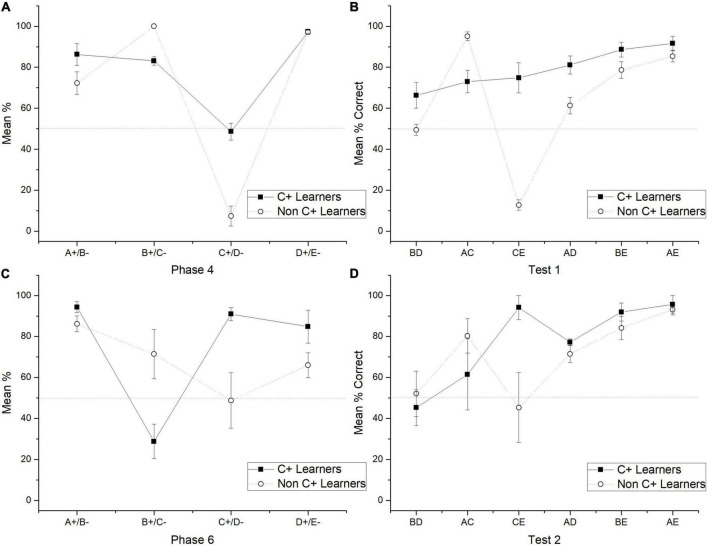
Mean percentage (SEM ±) of correct responses for C + learners and non C + learners in phase 4 **(A)**, test 1 **(B)**, phase 6 **(C)** and test 2 **(D)**.

**FIGURE 4:**
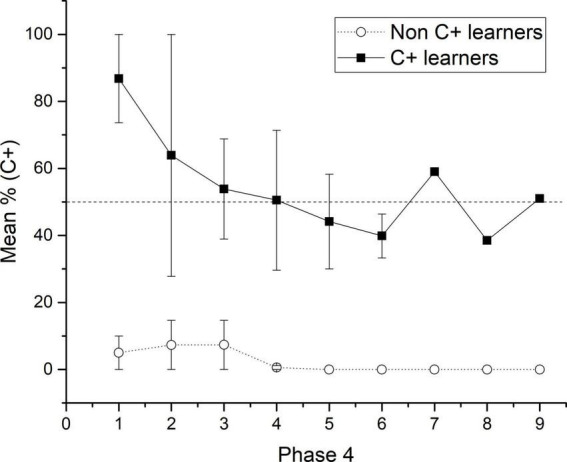
Mean percentage (SEM ±) of C + responses for C + learners and non C + learners across the nine sessions of phase 4.

The general results showed that the use of probabilistic outcomes affects the learning of premises so that the number of sessions for reaching the criterion tends to increase and the amount of subjects unable to reach the criterion also increases (see below the analysis of individual differences). The effects on learning premises seem to be focalized during C + D- premise, where most subjects could not reach the criterion. The general performance deficits in C + D- premise are associated with performance deficits in the crucial pair BD and CE during the test. Those deficits could not be corrected even after overtraining of C + D- premise during phase 5. However, there was a marginal improvement, so during phase 5, C + performance did not differ from chance, whereas before phase 5, it was below chance. Moreover, after phase 5, the CE pair did not form chance during test 2, whereas before phase 5, it was below chance levels. Consequently, performance deficits in C + D- seem to be associated with performance deficits in BD. Subjects who reached (two subjects) and did not reach the criterion (five subjects) during C + D- were analyzed separately. For abbreviation purposes, the formers were labeled as “C + learners” and the latter as “non-C + learners.” The differences in performance were noticeable since phase 4, where for C + learners, C + retained better performance than non C + learners across the nine sessions of phase 4. Differences in performance can be seen since phase 4, where the most noticeable differences were that C + learners sustained a C + performance close to chance levels (*M* = 48.57), whereas non C + learners sustained a C + performance below chance levels (*M* = 7.33) (see Figures 3A, 4), C + learners outperformed non C + learners in A + (*M* = 86.22 vs. *M* = 72.24), but not in B + (*M* = 83.07 vs. *M* = 100). D + premise remained with similar performance for C + learners (*M* = 97.35) and non C + learners (*M* = 97.11).

During test 1, C + learners showed better performance in all non-adjacent pairs than non C + learners (except for AC) (see Figure 3B). The most important difference is on the BD pair, where C + learners reached above chance levels, whereas non C + learners did not. During phase 5, C + learners reached a mean performance of 92.62%, whereas non C + learners only reached 39.9%. During phase 6, C + learners experienced a noticeably low performance in B + (*M* = 28.8) compared to non C + learners (*M* = 71.4). Additionally, the former reached high levels of performance during C + (*M* = 90.95) and the latter kept chance levels performance (*M* = 48.77). For the A + subgroup, performances were similar (C + Learners *M* = 94.32, non C + Learners *M* = 86.17). For the D + subgroup, C + learners performed slightly better (*M* = 84.8) than non C + learners (*M* = 66.02) (see Figure 3C).

In test 2, the apparent advantage acquired by C + learners seemed to disappear, so their performance was almost indistinguishable from non C + learners. Moreover, the former had an unexpected asymptotic performance during CE (*M* = 94.1), whereas the latter remained in chance levels performance (CE, *M* = 45.32). Therefore, aside from the mentioned differences in the CE pair, both subgroups had a similar performance, where more extreme pairs are better solved than more central pairs (see Figure 3D).

The analysis of individual differences confirms the general finding about the possible association between C + D- pair and BD performance. So, C + learners showed a better performance in the crucial pair BD and CE pair compared to non C + learners in test 1. This association seems to disappear in test 2, where both subgroups showed similar performance (except for the CE pair). The explanation of these differences requires further examination.

## Discussion

The main findings can be summarized as follows: (a) the use of probabilistic reinforcement seems to be associated with a delayed acquisition criterion and performance deficits in specific premises (e.g., C + D-), (b) the specific deficit in C + D- premise, remains across the entire procedure, and it seems to prevent the expected BD preference during both tests. However, a pattern similar to an SDE was still retained (c) when C + D- was overtrained, the expected SPE is affected. So, C + improved to chance levels, whereas B + decreased to chance levels, (d) for test 2, TI is still absent. However, CE marginally improved until reaching chance levels. A pure associative account or a pure positional ordering-based account is not enough to explain these findings. Instead, some effects can be explained by an associative account and others by a positional ordering account.

Previous studies have employed probabilistic reinforcement as a way to control value transfer. For example, Weaver et al. (1997) found evidence of TI despite imposing a 0.5 probability of reinforcement in both extreme pairs (A ± B- and D + E ±). On the other hand, other studies have employed a 0.5 probability of reinforcement to confirm value transfer effects (Zentall and Sherburne, 1994). The rationale behind those manipulations is that a 0.5 probability reduces the amount of value transferred from the S + to the S-. Regarding that assumption, if there was value transfer involved in TI procedures, in the current experiment that variable was controlled since the probabilities associated with S + and S- were kept constant and were always higher for S + than for S-. Besides, because a probability of 0.5 would not allow discrimination between premises, a probability of 0.8 was imposed on the S + and a probability of 0.2 for the S-. So, both S + and S- can be differentiated from a truly random contingency of *p* = 0.5. Regarding that S + and S- are associated with probabilistic reinforcement, they are not strictly “positive” or “negative.” In spite of that, the arrangement employed should have allowed an ordering A + B-, B + C-, C + D-, and D + E, where the first stimulus is consistently the most reinforced and the last stimulus is the least reinforced. The most direct and recent approach to the effect of probabilistic reinforcement on TI is the work of Jin et al. (2022). They found that monkeys (*n* = 3) can order stimuli sets even when probabilistic reinforcement in all premises is employed, which can be accounted for by the *Q*-learning model, at least in their learnable set. Despite the similarities with our study, they do not report analysis of acquisition, neither specific details about performance for each non-adjacent pair nor differences from chance in the BD pair. Consequently, our findings cannot be compared with theirs.

Along with the above mentioned, there is previous empirical evidence showing that subjects can respond differentially to probabilistic Pavlovian contingencies (Rescorla, 1968; Wasserman, 1974). Therefore, in the current experiment, TI was expected, although with delayed acquisition due to the complexity of the task.

### Group performance

The first main finding was the sudden lowering in performance in phase 3. Other studies in serial learning have found difficulties learning the third element of a series A→B→C→D, where “→” denotes what stimulus follows after responding to the previous one and responding to the last one is reinforced, being C the worst stimulus solved (Straub and Terrace, 1981). Not only was stimulus C the worst solved, but also the corresponding training phase A→B→C was the one when the failures to reach criterion started (70% of correct responses as criterion). Accordingly, during phase A→B, all subjects reached the criterion, during phase A→B→C, one subject failed. During phase A→B→C→D two subjects failed. Wynne (1997) reported five out of eight subjects failing to reach criterion. Two subjects failing to reach criterion in phase 2 and other three failing in phase 4. Phase 2 and phase 4 they both contained all premise pairs.

More recent studies in TI have not reported such as dropout ratio in pigeons (five out of seven) during premise pair C + D- (Lazareva and Wasserman, 2006, 2012; Camarena et al., 2018; Zentall et al., 2019), but those procedures used probabilities of 0 and 1, which makes the discrimination easier than the one employed in our experiment. Other manipulations reported dropouts in subjects’ performance, for example, von Fersen et al. (1991) used six pigeons exposed to all premise pairs in experiment one, but before the test, two subjects were excluded because they did not reach the 60% criterion. In the third experiment of the same study (von Fersen et al., 1991), it was reported that one subject ceased responding when the closed series was arranged. According to the above mentioned, the found deficits in C + D- and the dropout rate could be attributed to increased difficulty imposed by the probabilistic reinforcement. This trend can also be found in relatively easier procedures, for example, in the ambiguous cue procedure, only three cues are involved: P as S +, N as S-, and A as the ambiguous cue. A is always reinforced in presence of N but never reinforced in presence of P. Under this arrangement, when P is partially reinforced (*p* = 0.5), performance drops in PA trials below chance levels, but when training is extended several sessions, PA discrimination can reach above chance discrimination (Vasconcelos and Monteiro, 2014 in starlings; García-Leal et al., 2017 in pigeons). Therefore, regarding the evidence mentioned above, (a) can be explained as a consequence of C + D- being one of the most difficult pairs to solve in a five stimuli series, this pair became even more difficult when probabilistic reinforcement was introduced.

In relation to (b), the lowering in performance in C + D- can also be attributed to the increased difficulty of the task, since this lowering in performance is expected according to the SPE (see von Fersen et al., 1991; Wynne, 1995). As a result, C + D- performance becomes even worse than the expected SPE, when probabilistic reinforcement is introduced. Besides C + D- performance during phase 4, B + and D + showed almost the same asymptotic levels of performance. This could explain the lack of preference for B over D during both tests, which could imply the involvement of the over expectation effect (Rescorla, 1970). However, this possibility deserves further verification, since it is not clear if over expectation could provoke chance performance, over-chance performance, or below chance performance. In the case of CE performance, there seems to be a floor effect, which provoked below chance levels performance during test 1. Since C + had the lowest associative strength and E- was always reinforced with a 0.2 probability, when CE is presented during test 1, subjects are responding to the stimuli with the lowest associative strength. Regarding the performance in phases 5, 6, and test 2, the above-mentioned effects seem to be changed by a blocking effect, this would explain (c). Consequently, after C + D- was overtrained in phase 5, C + improved to chance levels, but B + decreased to chance levels in phase 6. Therefore, during phase 6, B + and C + did not differ from chance, whereas A + and D + differed from chance. Additionally, B + did not differ significantly from D +. Under this regard, the associative strength gained by C + seems to be equal to the associative strength of B + and D + (see Figure 2C) by blocking B + C- and D + E- performances, whereas A + remained almost the same. In accordance with this pattern, during test 2, BD performance again did not differ from chance, whereas CE reached chance levels (as an attenuation of the previous floor effect). This blocking effect would explain (d).

As can be seen, the main difference between test 1 and test 2 is the improvement in CE performance. Aside from that, the pattern resembles the expected SDE where the most central pairs are worse solved compared with extreme pairs. This finding suggests that even when performance has been noticeably impaired during training (including the expected SPE), subjects retain some ordering during the test, which cannot be entirely explained by differences in associative strength. This is the case for BD performance, which did not differ from chance in both tests. The performance during BD, cannot be always predicted from previous training, since in phase 4, B + and D + reached the same asymptotic performance. However, in phase 6, B + did not differ from D +, but B + remained at chance levels, whereas D + reached above chance levels. If B is at chance levels and D above chance levels, a D > B preference would be expected, which did not happen. These findings suggest that subjects still perform during BD as being the most difficult to solve. However, the data from individual differences showed that BD can be correctly solved even with probabilistic reinforcement administered.

### Individual differences

Due to the reduced sample size (*n* = 7), all the statements about individual differences remain as mere descriptive statements, which is why statistical comparisons between subgroups were omitted. Nevertheless, regarding the number of trials administered (see below the absolute frequencies) and the relatively stable performance patterns obtained, we still hold that performance in C + D- is a reliable predictor for performance during the test.

Several cases of individual differences can be found in TI procedures. For example, Piaget (1921) reported 2 out of 37 are capable of solving their verbal TI task, those who were capable of forming the relative categories ‘‘*brune, blonde et blond claire*’’^2^ (Piaget, 1921, p. 162). Lazareva and Wasserman (2010) reported individual differences in human performance dependent on the level of awareness of the task, having the subjects with a better understanding of the stimuli hierarchy with the best performance during the test compared with those who did not understand the hierarchy. Additionally, they reported a dropout of 96 participants from 166 because of deficits in performance. More recently, Lazareva et al. (2015) reported individual differences in pigeons, those differences were determined by their performance after the bias reversal phase. Accordingly, subjects who preferred B over D after bias reversal was called the *relational group*, whereas those who preferred D over B were called the *associative group*. This distinction addresses the fact that subjects can solve the task by regarding the associative strength of the stimuli or by regarding their ordinal position. In the case of the present findings, there is no relational or associative group. Instead, there is a group that can reach the criterion during C + D- (phase 3) and another that cannot (C + learners and non C + learners, respectively). Both groups seem to respond to the ordering of the stimuli and their associative strength. However, C + learners seem to have a general performance advantage in that ordering.

In order to address the found individual differences by an associative account, we calculated the ratio of correct responses for each subject and stimulus. We divided the reinforced choices by the non-reinforced choices of each stimulus (e.g., B + /B-). In the case of extreme stimuli such as A +, the frequency of choices of A + was divided by the frequency of choices of B-, and absolute frequencies were also reported. This approach has been previously used in pigeons (Lazareva and Wasserman, 2006).

As can be seen in Table 2, before test 1, there is a noticeable difference between C + learners and non C + learners, where the ratios for C + are higher for the former and lower for the latter. The subject P03 is a special case since it is close to the criterion during phase 3. In fact, it reached the criterion during phase 4, but this did not translate into better performance during test 1 or test 2. Instead, subject P03 behaves like a non C + learner. Besides, B values are higher than D values in both subgroups, which would predict a B > D preference.

**TABLE 2:** Correct responses ratio for each subject before test 1.

Correct choices ratio before test 1							
Subject	P01	P02	P03	P05	P06	P07	P08
A +	5.16	4.07	2.74	2.09	3.56	4.05	5.31
B +	3.54	2.81	2.11	1.64	2.80	4.09	4.19
C +	0.00	0.00	14.36	0.00	5.82	9.54	0.43
D +	0.26	0.28	0.17	0.30	1.58	0.41	0.29
**Absolute frequencies for correct and incorrect choices before test 1**							
A +	903	912	973	862	1,120	405	961
B –	175	224	355	412	315	100	181
B +	620	630	748	676	883	409	758
C –	0	140	11	0	133	70	7
C +	0	0	158	0	574	668	3
D –	1,411	1,445	972	1,415	226	658	1,407
D +	368	404	170	429	362	270	411
E –	8	45	8	3	88	31	45

When phase 4 was administered, C + learners sustained higher performance for the C + pair during each session compared with non C + learners. This trend was maintained until the end of phase 4 (see Figure 4). Therefore, according to the acquired associative strength at the end of phase 4, all subjects should prefer B over D during test 1.

**FIGURE 5:**
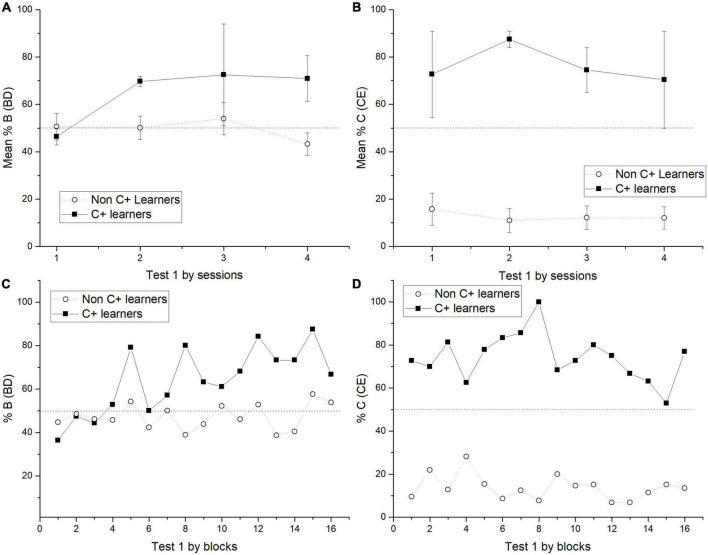
Mean percentage (SEM ±) of correct responses in BD across sessions during test 1 for BD **(A)** and CE **(B)**, including C + learners and non C + learners. Block analysis for BD preference in test 1 includes both subgroups, each block contains 50 trials. So that, the X axis includes 16 blocks of 50 trials taken from four test sessions **(C)**. Block analysis for CE preference in test 1 **(D)**.

When test 1 was administered, C + learners and non C + learners differed across sessions. Therefore, the expected B > D preference increased for C + learners, whereas for non C + learners, it remained close to chance levels. In the case of the CE pair, C + learners outperformed non C + learners across all test sessions (see Figures 5A–D).

Because of the found trend in BD performance, test sessions were analyzed by blocks of 50 trials. This analysis revealed that it was at the end of session 1 when C + learners started to have a preference for B > D. After that, the preference remained relatively stable (see Figure 5C). This outperformance from C + learners is consistent with the reported correct response ratios.

**FIGURE 6:**
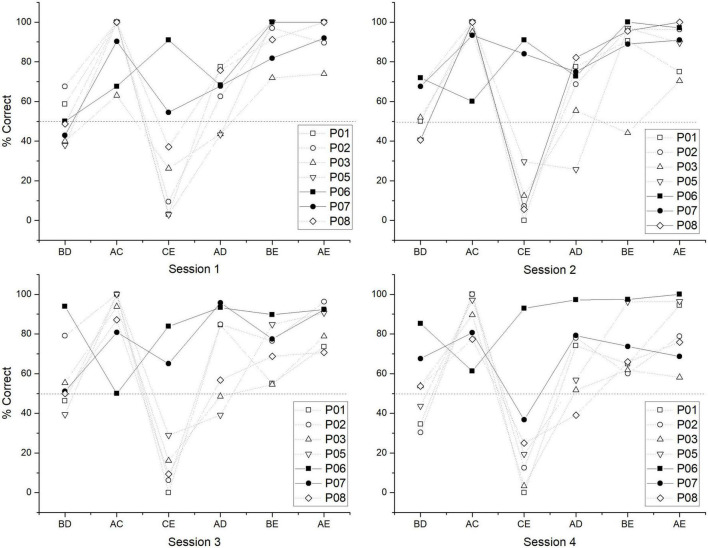
Individual performance for each test session of test 1. Closed symbols correspond to C + learners and open symbols to non C + learners.

In order to find other trends between subgroups, individual performance for each test session was plotted. As can be seen, there is a relatively stable pattern where non C + learners have close to chance values in BD, below chance values in CE, and asymptotic values in AC, whereas in C + learners, there is a trend of having above chance values in BD, higher values for CE, and lower but above chance values in AC (Figure 6).

After overtraining (phases 5 and 6), it was expected an improvement for non C + learners. This improvement was found but it did not allow the expected B > D preference. Thus, when test 2 was administered, C + learners still had a slightly different performance compared to non C + learners. Consequently, C + learners’ performance in BD, showed values close to chance levels similar to non C + learners, AC performance was similar between subgroups, whereas, for CE performance, C + learners still had higher performance than non C + learners. However, in this case, non C + learners sustained some values with above chance levels, which never happened during test 1. This trend is consistent with Table 3, since, for most non C + learners, C + ratios are higher than before test 1. Nevertheless, BD performance still cannot be predicted from correct response ratios, since for most cases, B values are higher than D values. Consequently, during test 2, both subgroups’ performances became similar, being the most noticeable difference in the CE performance.

**TABLE 3:** Correct responses ratio for each subject before test 2.

Correct choices ratio before test 2							
Subject	P01	P02	P03	P05	P06	P07	P08
A +	8.44	3.29	46.00	5.31	14.50	18.80	5.56
B +	10.89	4.04	33.00	4.50	7.50	3.20	0.33
C +	0.00	1.00	13.41	15.09	6.89	5.63	4.64
D +	0.16	0.19	0.33	0.43	2.18	2.21	0.36
**Absolute frequencies for correct and incorrect choices before test 2**							
A +	76	79	92	85	87	94	100
B –	9	24	2	16	6	5	18
B +	98	97	66	72	45	16	6
C –	0	1	27	23	65	84	76
C +	1	1	362	347	448	473	353
D –	426	492	153	160	44	33	153
D +	67	94	50	68	96	73	55
E –	33	12	48	29	9	22	39

Regarding the above mentioned, the reported main effects still hold. Thus, the trend between both subgroups can be accounted for Pavlovian mechanisms such as blocking. Nevertheless, the positional ordering of the stimuli, cannot be accounted for Pavlovian mechanisms. Particularly, the BD performance.

As can be seen, during test 1, C + learners performed better than non C + learners in all non-adjacent pairs, except AC pair. This exception can be explained by the fact that, when AC is presented, the contrast between stimuli is greater for non C + learners than for C + learners, since, for non C + s learners, the associative strength of C is extremely low. This contrast also applies when CE is presented, which is why CE is better solved for C + learners than for non C + s learners because the contrast between C and E is greater for C + learners. These differences in associative strength in C + can account for test 1 performance. The most important difference between both groups is the fact that C + learners developed a B > D preference during test 1, whereas non C + learners did not. This performance during the crucial pair BD cannot be accounted for associative differences, since for C + learners, B + remained slightly below D +, while for non C + learners, B + and D + seemed to have reached asymptotic levels (see Figure 3A). Therefore, for the former, a D > B preference would be expected and for the latter, a BD preference at chance levels would be expected.

Thus until test 1, (a) is attenuated in C + learners because they passed C + D- pair and maintained close to chance levels performance in C + during phase 4; (b) is absent because they reached a B > D preference, but some sort of SDE remained.

**FIGURE 7:**
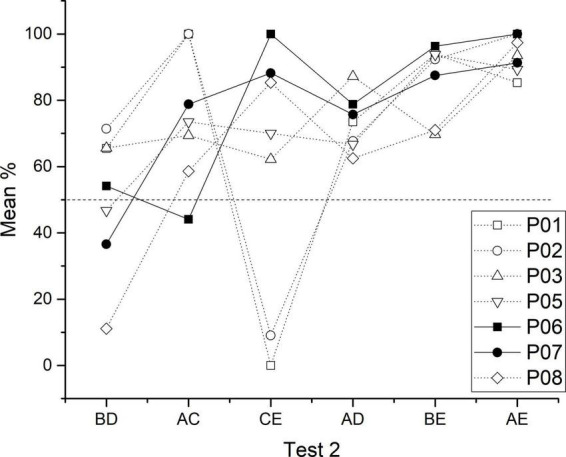
Individual performance for each test session of test 2. Closed symbols correspond to C + learners and open symbols to non C + learners.

Afterward, when over training in C + D- was administered in phase 5, C + learners reached 92.62% of the correct response, while non C + learners only reached 39.9%. This difference seems to affect the performance during phase 6. As a result, the high associative strength in C + learners blocked the B + C- and D + E- performance, leading B + to below chance levels and increasing C + to an asymptotic level, whereas in non C + learners, B + reached above chance levels, C + did not differ from chance (improved), and D + decreased near to chance levels. In both subgroups, A + performance remained almost the same, whereas D + had higher performance in C + learners than in non C + learners (see Figure 3C). As can be seen, because in non C + learners, B + and D + stimuli, both had very similar performance, a BD performance at chance levels would be expected, whereas in C + learners, B + performance was noticeably lower than D + performance, therefore a D > B preference would be expected. In the second test, both groups showed an ascending pattern similar to the expected SDE (except for the CE pair). However, performance during the BD pair remained at chance levels with very similar percentages in both subgroups. In the case of the CE pair, both subgroups increased their performance compared to test 1, C + learners reached asymptotic levels, whereas non C + learners improved only to chance levels. Therefore, test 1, as well as test 2, showed that performance during the crucial pair BD cannot be predicted by the associative strength acquired by each stimulus in the previous phase. Therefore, effects (c) and (d) depend on the subgroup. Accordingly, when overtraining was administered in C + learners, B + performance remains lower than D + performance, because the asymptotic C + performance blocked B + C- performance (see Figure 3C), whereas, in non C + learners, C + just reached chance levels, keeping B + and D + at similar performance. In the case of (d), for C + learners, B > D preference was lost during test 2, whereas non C + learners performed as in test 1. The CE improvement was noticeably larger for C + learners than for non C + learners (see Figure 7).

The main findings raise several questions about what is learned during the training of all premises and the overtraining of particular premises. First, learning C + D- at the chance levels would imply no discrimination between stimuli, which by making C = D would change the expected ordering A > B > C > D > E leading to A > B > C = D > E, which in turn would imply C > E during test, a preference that was only found in C + learners. Therefore, it is unclear what is exactly learned by the subjects when the performance in a particular premise does not differ from chance. Second, it is unclear why only two sessions of over training in C + D- unexpectedly affect phase 6 performance and test 2 performance in a different way for each subgroup. This finding implies that there is an optimal level of C + D- performance required to successfully obtain TI, this optimal level clearly depends on the previously associative strength acquired by C +. However, it is not clear if the required level of performance is acquired during C + D- or during all premises presentations. The performance of subject P03, suggests that it is phase 3 that determines the differences in the test. Third, equal performance for B + and D + appears to be associated with chance levels of performance during the BD pair. However, the same at chance level performance can be obtained when B + has lower performance than D +.

Further studies involving probabilistic reinforcement should address which is the optimal way to administer C + D- pair. There seem to be at least two options, including correction trials during phase 3 or training D + E- as a separated pair before all pairs are presented in phase 4. The latter option could reduce the possible blocking effect from C + to D + when subjects reach asymptotic levels of performance during C + D-. Additionally, the analysis by blocks during the test seems to be helpful in revealing the possible effects of repeated exposure to several session tests. This analysis by blocks also suggests that B > D preference might not occur at the beginning of the test sessions. This is important since most of the studies apply one single test session or average all test sessions (e.g., Wynne, 1997; Lazareva and Wasserman, 2006). Finally, it would be interesting to know if mathematical models applied in primates (e.g., Jin et al., 2022) can account for pigeons’ performance and the blocking and over expectation effects, such as the ones reported here.

In summary, regarding all subjects, test performance cannot be explained completely by associative strength or by logical ordering. Instead, subjects seem to have a disrupted performance for pair involving C during both tests, but despite the disruption, they still managed to order the remaining stimuli similar to an SDE. Therefore, even when probabilistic reinforcement disrupts C + D- learning during training, subjects still retained the positional ordering for the other stimuli, this is evident when CE performance drops below chance in test 1 but raises at chance levels during test 2, retaining a pattern similar to an SDE. On the other hand, the fact that individual differences appeared specifically in C + D- pair requires further verification since there is no associative or logical account for that effect and it has not been reported before.^3^ Despite these individual differences, Pavlovian contingencies seem to be affecting the task. Thus, during test 1, C + learners have a clear advantage in solving all non-adjacent pairs compared to non C + learners. However, during test 2, this advantage disappears for C + learners, apparently because, when C + reaches asymptotic levels during overtraining, it blocks B + C- and D + E- performance in phase 6 and prevents B > D preference. For non C + learners, overtraining C + D- does not improve BD performance in test 2. Overall, this finding suggests that an optimal value of associative strength during C + D- is required in order to learn TI. It remains unclear why BD preference is not always predicted by B + and D + performance when all premises are presented (phase 4 and phase 6). For example, studies in pigeons that do not use probabilistic reinforcement (e.g., Camarena et al., 2018) show that B > D preference remains despite B + performance being lower or equal to D + performance when all premises are presented, whereas in the present experiment, B > D preference was not obtained when performance during B + and D + they were both equal regarding all subjects. However, B > D preference was found in some subjects even when B + was slightly lower than D +.

## Data availability statement

The raw data supporting the conclusions of this article will be made available by the authors, without undue reservation.

## Ethics statement

This animal study was reviewed and approved by the Local Ethical Committee of the Center for Studies and Investigations in Behavior, by the University of Guadalajara.

## Author contributions

HC and OG-L wrote the manuscript. ZS-H and EB ran the experiment to obtain the data. All authors contributed to the article and approved the submitted version.

## Footnotes

1 Piaget basically refers to a child misunderstanding of the fact that if Edith is fairer than Suzanne and Edith is darker than Lili, it implies that Edith is fairer and dark at the same time. When in fact the logical order implies that Suzanne is darker than Edith and Edith is darker than Lili.

2 These categories can be understood as “brunette, blonde and blonde pale”. Those categories could not be formed for the other subjects, since they understood the task as described in foot note 1.

3 We have found the same trend using probabilities of 0.7 and 0.3 for the S + and S- stimuli, respectively (unpublished data).
